# Impacts of dietary animal and plant protein on weight and glycemic control in health, obesity and type 2 diabetes: friend or foe?

**DOI:** 10.3389/fendo.2024.1412182

**Published:** 2024-07-31

**Authors:** Javad Anjom-Shoae, Christine Feinle-Bisset, Michael Horowitz

**Affiliations:** ^1^ Adelaide Medical School and Centre of Research Excellence in Translating Nutritional Science to Good Health, University of Adelaide, Adelaide, SA, Australia; ^2^ Endocrine and Metabolic Unit, Royal Adelaide Hospital, Adelaide, SA, Australia

**Keywords:** animal protein, appetite, food intake, gastrointestinal function, glycemic control, obesity, plant protein, type 2 diabetes

## Abstract

It is well established that high-protein diets (i.e. ~25–30% of energy intake from protein) provide benefits for achieving weight loss, and subsequent weight maintenance, in individuals with obesity, and improve glycemic control in type 2 diabetes (T2D). These effects may be attributable to the superior satiating property of protein, at least in part, through stimulation of both gastrointestinal (GI) mechanisms by protein, involving GI hormone release and slowing of gastric emptying, as well as post-absorptive mechanisms facilitated by circulating amino acids. In contrast, there is evidence that the beneficial effects of greater protein intake on body weight and glycemia may only be sustained for 6–12 months. While both suboptimal dietary compliance and metabolic adaptation, as well as substantial limitations in the design of longer-term studies are all likely to contribute to this contradiction, the source of dietary protein (i.e. animal vs. plant) has received inappropriately little attention. This issue has been highlighted by outcomes of recent epidemiological studies indicating that long-term consumption of animal-based protein may have adverse effects in relation to the development of obesity and T2D, while plant-based protein showed either protective or neutral effects. This review examines information relating to the effects of dietary protein on appetite, energy intake and postprandial glycemia, and the relevant GI functions, as reported in acute, intermediate- and long-term studies in humans. We also evaluate knowledge relating to the relevance of the dietary protein source, specifically animal or plant, to the prevention, and management, of obesity and T2D.

## 1 Introduction

**Figure 1:**
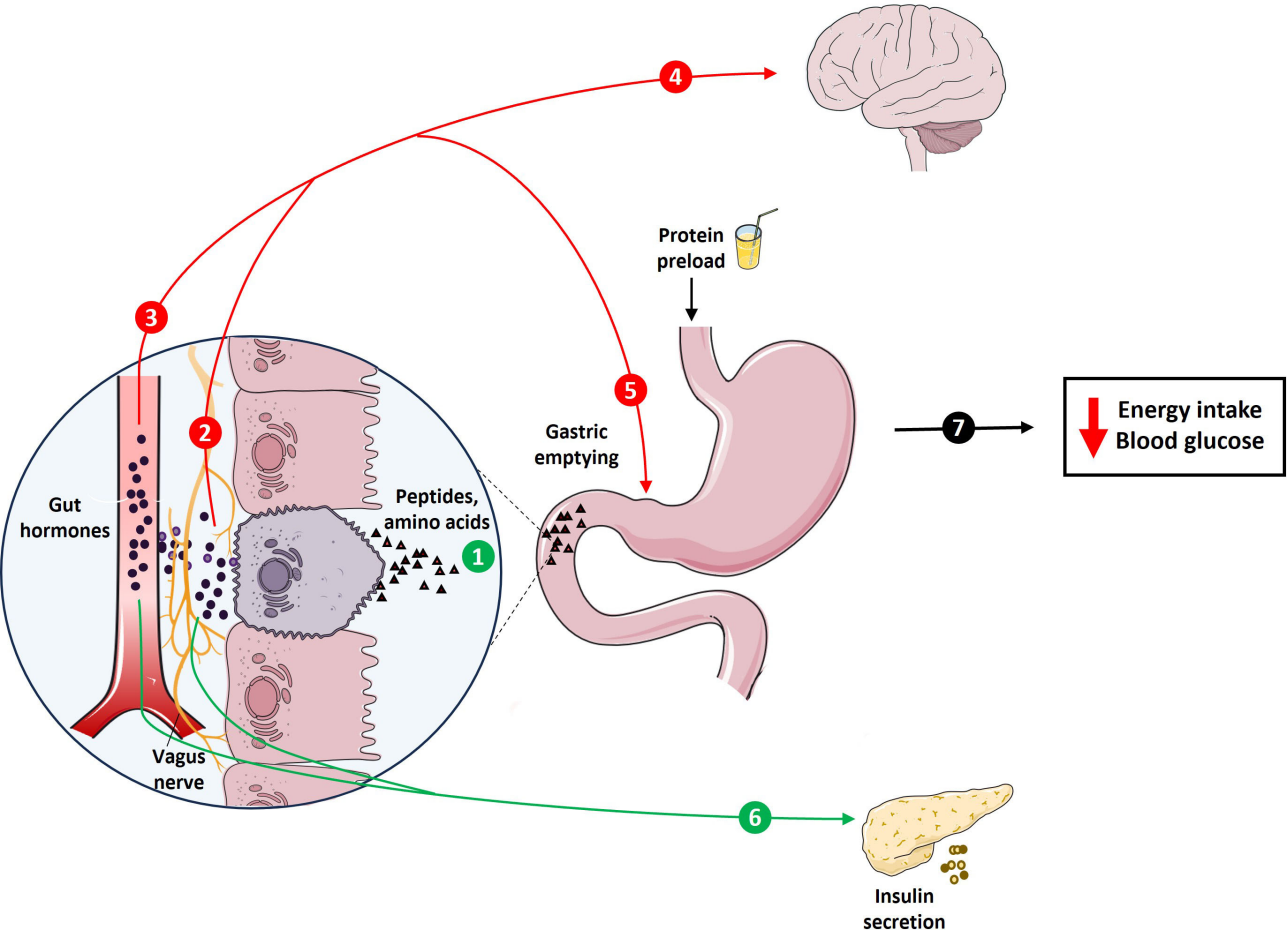
Schematic representation of protein-induced stimulation of gastrointestinal (GI) functions, including GI hormone release and slowing of gastric emptying, which are integral to the regulation of energy intake and glycemia. The presence of protein digestion products, including peptides and amino acids, in the GI lumen stimulates key GI hormones, including CCK, the incretins, GIP and GLP-1, and PYY (1). These hormones exert their effects through various pathways, including activation of hormone-specific receptors on vagal afferent endings (2) or following transport through the bloodstream (3). These inputs, together, are conveyed to higher brain centers to modulate eating behaviors (4), as well as feedback regulation of GI motor functions, particularly stimulation of pyloric pressures, associated with the slowing of gastric emptying (5). GIP and GLP-1, when transported in the bloodstream and/or by activating receptors on vagal afferent endings, also stimulate insulin secretion from pancreas (6). Together, these signals contribute to the effects of protein to reduce energy intake and blood glucose (7).

In the last ~20 years, there has been substantial, and increasing, interest in promoting dietary protein intake to improve health outcomes (1–5). We believe that the first official recommendation for daily protein intake, reported in 1936 by the League of Nations (6), was 1.0 g/kg of body weight. This has been subsequently challenged by several joint Food and Agriculture Organization (FAO)/World Health Organization (WHO) expert committees, who made the current recommendation of 0.8 g/kg daily protein intake in healthy adults, accounting for ~10–15% of daily energy intake, in 2007 (7). There is now compelling evidence that high-protein diets, which can entail a protein intake up to 5-fold greater than the recommended daily amount, and are in most cases characterized by ~25–30% of energy intake from protein, facilitate weight loss and attenuate weight (re)gain, in individuals with obesity, and improve glycemic control in type 2 diabetes (T2D), in the intermediate-term, i.e. during 6–12 months’ consumption (8–10). Protein suppresses energy intake (11–16), and reduces postprandial glycemia (17–20). These effects may be attributable to the capacity of protein to stimulate both gastrointestinal (GI) hormones (11–13) and postabsorptive, possibly ‘central’, mechanisms in response to meals (21, 22). Key GI hormones include cholecystokinin (CCK), the so-called ‘incretin’ hormones, glucose-dependent insulinotropic polypeptide (GIP) and glucagon-like peptide 1 (GLP-1), as well as peptide tyrosine-tyrosine (PYY), which are pivotal to the regulation of both energy intake and/or postprandial blood glucose, in some cases, at least in part, through slowing of gastric emptying (23–25) (**Figure 1**).

In contrast to these potent acute/intermediate-term effects of protein, there is evidence that the beneficial effects of greater protein intake on body weight and glycemia may only be sustained for 6–12 months (26–28), which has been attributed to both suboptimal dietary compliance and metabolic adaptation. However, the substantial variations, as well as limitations in the design of longer-term studies, including inconsistencies in the amount and composition of tested foods, and the characteristics of study participants (e.g. age, ethnicity and race) are also likely to be relevant. There are also considerable variations in the source of dietary protein between individuals worldwide (29, 30), which may be derived from animal- and/or plant-based foods. This issue has received less attention despite compelling evidence that animal and plant proteins may have different metabolic effects in the longer-term (31–33). This issue has assumed increasing importance, particularly in view of emerging evidence derived from recent epidemiological studies to indicate an increased risk of T2D with animal, but a protective effect of plant, protein (34–36).

The focus of this review relates to the effects of dietary protein on appetite, energy intake and postprandial glycemia, and the relevant GI functions, including the stimulation of GI hormones and slowing of gastric emptying, as reported in acute, intermediate- and long-term studies in humans. We also evaluate knowledge relating to the relevance of the dietary protein source, specifically animal or plant, to the prevention, and management, of obesity and T2D. While dietary protein is also of importance to other areas, including muscle mass, particularly in elderly and malnourished people, these and other issues are not addressed.

## 2 Acute effects of protein on appetite, energy intake and postprandial glycemia

A number of studies have shown that acute oral administration of protein preloads, in doses ranging from 20–70 g, dose-dependently reduce hunger, and induce fullness, associated with suppression of energy intake at a subsequent meal, in both healthy lean individuals and those with obesity (11–16, 20, 37). A comprehensive meta-analysis comprising 49 trials, which investigated the acute effects of protein preloads on commonly used markers of appetite, revealed decreases in hunger, desire to eat, prospective food consumption, and an increase in fullness in both lean and obese participants (38). These effects were associated with a reduction in subsequent food intake, when participants were presented with a standardized meal (38). These effects of protein are also often accompanied by reductions in postprandial glycemia. Indeed, a higher protein intake, either as a ‘preload’ before, or as part of, a carbohydrate meal, has been shown to reduce postprandial glycemic excursions, in both lean and obese individuals with and without T2D (10, 13, 17–20, 39–42). Accordingly, the outcomes of these acute studies are consistent, showing that a higher protein intake at a meal has beneficial effects to reduce both energy intake and postprandial glycemia.

The acute appetite- and glucoregulatory effects of protein have been shown to vary between different sources of protein (43–59) (**Table 1**). For example, when the effects of preloads, containing either milk proteins (whey or casein protein), egg, turkey, tuna, or soy protein, were compared, each suppressed hunger and energy intake, but whey protein had the most profound effects (44, 49, 51, 53, 56). In contrast, a number of studies reported that whey protein was less satiating than some other proteins (46, 47, 55). For example, when the effects of whey protein, pea protein hydrolysate, a combination of whey protein and pea protein hydrolysate, and control milk protein (80% casein and 20% whey) were compared, pea protein hydrolysate was the most effective in suppressing hunger and desire to eat, with no difference in their effects on subsequent energy intake (46). Veldhorst et al. also reported that both alpha-lactalbumin and gelatin are ~40% more satiating than whey protein, inducing a related ~20% reduction in subsequent energy intake (55). However, when compared with casein or soy, whey protein was still more effective in suppressing energy intake (56). Milk proteins have also been found, in some studies, to exert more potent effects to reduce blood glucose than turkey, fish, egg, or pea proteins (52–54, 57). For example, when 45 g of protein, of different sources (either gluten, cod, casein, or whey), was added to a high-fat meal, the postprandial blood glucose response in T2D was less with whey, compared to the other proteins (52). In contrast, in another study in healthy and prediabetic adults, there was no difference in postprandial glycemic excursions between whey and casein, when added to a drink containing maltodextrin (50). Whether variations in the effects of different protein sources to reduce postprandial glycemia are associated with the magnitude of their effect on appetite remains uncertain, with some studies suggesting a strong relationship, particularly for whey (43, 53, 60). The latter is potentially attributable to the rapid digestion of whey protein, due to its high solubility in the acidic environment of the stomach, leading to the stimulation of GI mechanisms more effectively than other proteins (49, 60). However, comprehensive evidence comparing all types of protein sources, particularly different plant-based proteins, is lacking and further investigation is required.

**Table 1:** Acute effects of different protein preloads (animal vs. plant) on ad-libitum energy intake and postprandial blood glucose levels.

First author	Country	Design	Sample size (n)	Age (y)	BMI (kg/m^2^)	Health status	Protein source	Protein dose	Duration^1^ (min)	EI^2^ (kcal)	BG^3^	Ref
Hall et al. (2003)	UKD	Cross-over	16 (M/F)	22	21.7	Healthy lean	Casein Whey	48 g	90	1084 878	NR	(49)
Anderson et al. (2004)	Canada	Cross-over	13 (M)	22	22	Healthy lean	Egg Soy Whey	0.65 g/kg (~46 g)	60	912 729 661	NR	(44)
Nilsson et al. (2004)	Sweden	Cross-over	12 (M/F)	20-28	21.9	Healthy lean	Gluten Cod Cheese Milk Whey	18.2 g	90	NR	35.4* 43.9 39.3 19.3 21.8	(57)
Bowen et al. (2006)	Australia	Cross-over	72 (M)	50-56	23-30	Healthy lean and overweight	Soy Gluten Whey	50 g 51 g 51 g	180	766 718 769	5.8 5.9 5.9	(45)
Diepvens et al. (2008)	The Netherlands	Cross-over	39 (M/F)	42	27.6	Overweight	Pea Whey Pea + Whey	15 g	180	304 299 309	NR	(46)
Veldhorst et al. (2009)	The Netherlands	Cross-over	25 (M/F)	22	23.9	Healthy lean	Soy Casein Whey Gelatin Alpha-lactalbumin	25% of high-protein custard (~40 g)	180	767 736 687 556 501	122* 68 95 82 84	(55, 56)
Mortensen et al. (2009)	Denmark	Cross-over	12 (M/F)	64	28.9	T2D	Gluten Cod Casein Whey	45 g	480	NR	495* 396 375 233	(52)
Pal et al. (2010)	Australia	Cross-over	22 (M)	23	22.6	Healthy lean	Egg Turkey Tuna Whey	50.8 g	240	844 839 782 705	5.45 5.49 5.39 4.59	(53)
Acheson et al. (2011)	Switzerland	Cross-over	23 (M/F)	32	22.7	Healthy lean	Soy Casein Whey	0.81 g/kg (~56.7 g)	330	NR	5.9 6.1 6.1	(43)
Gunnerud et al. (2012)	Sweden	Cross-over	14 (M/F)	20-28	21.9	Healthy lean	Soy Whey	9 g	60	NR	60.6* 54.7	(48)
Teunissen-Beekman et al. (2014)	The Netherlands	Cross-over	48 (M/F)	58	28.6	Overweight or obesity	Egg Pea Milk	0.6 g/kg (~70 g)	240	NR	-3.8** -3.8 -4.2	(54)
Hoefle et al. (2015)	Germany	Cross-over	15 (M) 15 (M/F)	26 62	23.9 29	Healthy lean Prediabetes	Casein Whey Casein Whey	50 g	240	NR	5.7 5.4 7.5 7.7	(50)
Dougkas et al. (2018)	Sweden	Cross-over	28 (M)	28	23.4	Healthy lean	Plant proteins (oat, pea and potato) Milk proteins 50:50 mixture	25% of high-protein pudding (~25 g)	210	760 816 795	7.5 7.9 7.5	(47)
Melson et al. (2019)	USA	Cross-over	17 (M/F)	27	24.6	Healthy lean	Soy Whey	50 g 43.3 g	180	664 654	NR	(51)

M, Male; F, Female; Y, Years; BMI, Body mass index; EI, Energy intake; BG, Blood glucose; NR, Not reported; T2D, Type 2 diabetes.

^1^ Time interval between protein preload and an ad-libitum test meal; ^2^ Energy intake at an ad-libitum test meal; ^3^ Reported as peak concentration of postprandial glucose in mmol/L, otherwise indicated as areas under the curve (AUCs) (mmol/L.h*) or changes from baseline (mmol/L**).

### 2.1 Mechanisms underlying the effects of protein on energy intake and glycemia

The stimulation of both GI mechanisms, involving GI hormone release and slowing of gastric emptying (23–25), as well as post-absorptive mechanisms facilitated by specific circulating amino acids (21, 22), have been shown to be integral to these effects of protein. Protein, and its digestion products (amino acids), when administered directly into the GI lumen, stimulate key GI hormones, including CCK, the incretins, GIP and GLP-1, and PYY (61–70). In addition to the direct activation of receptors on submucosal vagal afferent and enteric neurons to modulate eating behavior (71), these hormones are transported in the bloodstream to affect peripheral organs, including the stomach, to stimulate pyloric pressures, which are important to the regulation of gastric emptying, and the pancreas, to stimulate insulin secretion (72), overall resulting in a reduction in postprandial glycemia (**Figure 1**). The rate of gastric emptying plays a key role in determining the postprandial glycemic response, particularly in the first 30–60 min following a meal, accounting for up to 35% of the variance in the initial glycemic response to a meal in healthy participants (73). With progressive impairment in glucose tolerance, this relationship exhibits a ‘shift to the right’, so that the 120-min blood glucose in a 75 g oral glucose tolerance test is inversely related to the rate of gastric emptying in healthy participants, but directly in people with T2D (74). Proteins also stimulate glucagon secretion, which may affect postprandial glycemia adversely (75). Moreover, postprandial glucagon secretion is characteristically exaggerated in individuals with T2D (76).

As alluded to, these acute effects of protein to modulate GI functions are dependent on the type of protein, with evidence that whey protein is more potent than other protein sources, including casein, fish, soy, gluten and pea protein (45, 46, 48, 49, 53, 56, 57). Accordingly, the majority of studies have focused predominantly on whey protein. For example, in healthy men, a 60-min intraduodenal infusion of whey protein, in loads of 0.5, 1.5 and 3 kcal/min, reflecting the physiological range of gastric emptying of ~1–3 kcal/min, has been shown to stimulate plasma CCK and GLP-1 concentrations, and pyloric pressures, all in a dose-dependent manner, associated with suppression of subsequent energy intake in both lean men (61) and those with obesity (62). At the highest load (3 kcal/min), whey protein also reduced blood glucose levels in individuals without T2D (61, 62). Oral preloads of whey protein, in doses of 30 and 70 g, also stimulated plasma CCK, GLP-1, glucagon, and slowed gastric emptying, associated with suppression of energy intake, and improved glycemia, in healthy men (13). In T2D, acute administration of whey protein, ingested as a preload, in a dose of 55 g, 30 min before a mashed potato meal, also stimulated GLP-1, GIP and insulin and slowed gastric emptying, associated with a substantial reduction in peak postprandial glucose of ~3 mmol/L (17). Moreover, these effects were shown to be sustained when whey protein (25 g) was given 30 min before each of three main meals, for 4 weeks (18). Similar acute effects of whey protein were evident when a preload incorporating whey (17 g) together with guar (5 g), a viscous polysaccharide that can itself reduce postprandial glycemic excursions, was given to individuals with T2D or prediabetes (77). 12 weeks’ treatment with this preload, consumed twice daily before breakfast and dinner in individuals with well-controlled T2D, had sustained effects to slow gastric emptying and reduce postprandial blood glucose (78).

There is evidence that the high content of branched-chain amino-acids (BCAAs), particularly leucine and isoleucine, in whey protein, contributes to its efficacy in reducing energy intake and glycemia, through stimulation of GI hormone secretion (57, 79, 80). For example, intraduodenal administration of L-leucine, in a load of 0.45 kcal/min (9.9 g over 90 min), stimulated CCK secretion and suppressed subsequent energy intake (63). Moreover, both L-leucine and L-isoleucine, when administered intragastrically, in a dose of 10 g, 30 min before a mixed-nutrient drink (500 kcal), lowered postprandial blood glucose (67). In contrast, valine was ineffective, potentially reflecting the concurrent stimulation of glucagon (67). L-leucine also stimulated C-peptide, a marker of insulin secretion, and both L-leucine and L-isoleucine slowed gastric emptying of the drink modestly (67). However, these effects of L-leucine and L-isoleucine were not evident in individuals with T2D for uncertain reasons (68). There is also compelling evidence that these effects of amino acids are type-specific, with some, such as L-proline (81), and L‐lysine (82), being less potent, compared to the aromatic amino acids, L-tryptophan (65, 66, 83) and L-phenylalanine (69). In a comparative analysis of the effects of four different amino acids (L-tryptophan, L-phenylalanine, L-leucine and L-glutamine) administered intraduodenally, L-tryptophan and L-leucine were shown to have the most potent effects to reduce energy intake, which was related to greater stimulation of plasma CCK (70). Both L-tryptophan (dose of 3 g) (66) and L-phenylalanine (dose of 10 g) (69), when administered intragastrically before a carbohydrate-containing drink, also lowered the blood glucose response in healthy lean participants (66, 69) and those with obesity (66).

The concept that these amino acids may also mediate the effects of dietary protein, after absorption, via both vagal mechanisms and direct effects on specific brain regions, including the hypothalamus and brainstem (21, 22, 84), was first introduced in 1956, as the so-called ‘aminostatic hypothesis’, which recognized that while amino acids are used primarily for protein synthesis, the amino acids remaining in the circulation might serve as a food intake-regulatory signal (84). BCAAs, particularly L-leucine, were shown to activate the mammalian target of rapamycin complex 1 (mTORC1), to act as a cellular fuel sensor in which hypothalamic activity is tied directly to the regulation of energy intake. In a variety of model systems, mTOR activity has been shown to be highly sensitive to plasma levels of L-leucine (85, 86). There is also emerging preclinical evidence to support a major role for BCAAs, particularly L-leucine and L-isoleucine, in β-cell signaling and metabolism, to acutely stimulate insulin secretion through activation of mTORC1, which is also responsible for increasing β-cell mass and function (87, 88). Elevated plasma concentrations of other amino acids, particularly L-tryptophan, which serves as a precursor for the neurotransmitter serotonin, a key regulator of appetite (89), have also been reported to be associated with reduced energy intake. A lesser number of studies have addressed the role of other amino acids. Both tyrosine and histidine can be converted into anorexigenic neurotransmitters, including dopamine, norepinephrine and histamine, but their contributions to protein-induced food intake suppression remain uncertain (90, 91). Thus, amino acids appear to mediate, at least in part, the effects of protein through distinct physiological pathways. This is likely to be important given that the amino acid composition of different sources of proteins may represent a major factor to account for their diverse metabolic effects in the longer-term.

## 3 Intermediate-term effects of protein on food intake, body weight and glycemia

The capacity of high-protein diets to induce weight loss has been examined primarily through two approaches; ‘ad-libitum’ diets, in which participants are allowed to consume based on their desire to eat, and ‘energy-restricted’ diets, where the proportion of protein is increased while restricting and then maintaining a constant total energy intake. Irrespective of the type of dietary protocol, in a majority of studies, enriching diets with a relatively high protein content has been shown to facilitate weight loss more than standard-protein diets (~10–15% of energy intake from protein) with intervention durations of up to 6 months (92–96). Ad-libitum high-protein diets, however, have shown more consistent efficacy, while under iso-energetic conditions, strict control of energy intake has invariably been associated with clinically relevant weight loss that compromised assessment of potential metabolic effects of protein. A number of meta-analyses have reported favorable effects of high-protein diets on weight loss (8, 9, 97). For example, a meta-analysis of 24 randomized clinical trials that only compared energy-restricted isocaloric high-protein (27–35% protein) and standard-protein (16–21% protein) diets, with a mean diet duration of 12 weeks, revealed modestly greater reductions in weight (-0.79 kg) and fat mass (-0.87 kg) with a high-protein diet (8). Another meta-analysis of 74 randomized controlled trials, investigating the effects of high-protein diets with or without energy restriction, with a mean duration of 6 months, also found reductions in body weight (-0.36 kg), body mass index (-0.37 kg/m^2^), and waist circumference (-0.43 cm) in the high-protein (16–45% protein) compared to the standard-protein (5–23% protein) diet group (97).

In contrast to the promising and relatively consistent outcomes of the shorter-term effects (≤6 months duration) of high-protein diets on weight loss in numerous studies, the majority of longer-term studies (at least 12 months in duration), albeit much fewer in number, found no effect of higher protein intake (26–28, 98–102). For example, in a follow-up to an intensive 6-month weight-loss trial, Due et al. reported that, at 12 months, weight loss was no greater in participants assigned to a high-protein diet (30% protein), compared with a medium-protein diet (12% protein) (98). A 2013 meta-analysis, which included 15 trials, in which the intervention period was for a minimum of 12 months, also revealed neither a beneficial, nor detrimental, effect of higher protein intakes on weight loss (28). In contrast, in a 12-month study, McAuley et al. reported modestly improved weight-loss maintenance (-6.6 kg) with a higher-protein diet (30% protein) than either a high-carbohydrate diet (-4.4 kg) or a high-fat diet (-5.5 kg), each containing 15% protein (99). Clifton et al. also found a direct relationship between weight loss and protein intake when comparing high-protein (34% protein) with high-carbohydrate diets (containing 17% protein) for 12 months, however, there was no difference in weight loss effects of the two diets (101). In two trials by Brinkworth et al, one in people with T2D (27), the other in normoglycemic individuals with obesity (26), the effects of a high-protein diet (30% protein) and a standard-protein diet (15% protein), both low in fat, during 8 to 12 weeks of energy restriction and 12 months of energy balance were compared, reporting a net weight loss in both groups, which was slightly greater in the high-protein group (-3.7 to -4.1 kg) compared with the standard-protein group (-2.2 to -2.9 kg). Accordingly, while the majority of evidence indicates that the efficacy of high-protein diets is attenuated in the longer-term, adherence to such diets may still facilitate weight maintenance, for at least up to 12 months (103). In addition, it should be noted that the interpretation of these studies is, in many cases, compromised by poor compliance and high dropout rates, precluding definitive conclusions regarding the long-term effects of high-protein diets on weight loss. However, there is unequivocal evidence that a progressive decrease in adherence is very common with any dietary intervention and, not surprisingly, irrespective of the macronutrient composition, a greater adherence to any energy-restricted diet is associated with a greater weight loss at both one (104) and two years (105). An inherent challenge in longer-term studies is to minimize the impacts of potential cofounders, including the unavoidable lack of blinding, as well as differences in participant characteristics (age, ethnicity and race), which may impact on the GI-induced effects of protein (106, 107).

While there is a lack of definitive evidence regarding the optimal dietary approach for T2D management, in the majority of cases, weight loss represents a primary strategy for improved glycemic control, usually assessed by measurement of glycated hemoglobin (HbA_1c_). In individuals with prediabetes or newly diagnosed T2D, a modest (5–10%) reduction in body weight improves glycemic control significantly (108). Accordingly, high-protein diets, because of their established weight loss effects, at least in studies of up to 6 months duration, have been advocated as a strategy to improve glycemic control (109). In a recent network meta-analysis of 42 randomized controlled trials, involving 4,809 patients with T2D, comparing the impacts of 10 different dietary approaches on glycemic control, high-protein diets were shown to be among the most effective in reducing both HbA_1c_ and fasting glucose (110). The beneficial effects of high-protein diets to reduce the postprandial blood glucose response, which, in individuals with relatively well-controlled T2D (i.e. baseline HbA_1c_ ≤8.0%), is the major determinant of glycemic control, have been reported in several trials (10, 111–113). However, there are also inconsistent observations, particularly in studies with duration of >6 months (26–28, 114–116). A 2012 meta-analysis, summarizing nine clinical trials with intervention durations between 4 to 24 weeks, revealed a modest, but significant, reduction of 0.52% in HbA_1c,_ but not fasting glucose, in individuals with T2D following a high-protein diet (~25–32% of energy intake) (9). However, no significant effects, on either HbA_1c_ or fasting glucose, were evident in a more recent meta-analysis of 13 trials with intervention durations ranging from 12 weeks to 52 months (116), although, given the large variations in study conditions, this is probably not surprising. Another meta-analysis, which included 15 trials with longer intervention durations (at least 12 months) in individuals with or without T2D, also found no effects on either HbA_1c_ or fasting glucose (28). Therefore, it remains uncertain whether sustained adherence to a high-protein diet improves glycemic control in T2D or prediabetes. It is also not known whether the positive outcomes of shorter-term trials reflect the use of protein per se, the concurrent reduction in weight, or both, particularly since these trials were often based on energy-restricted high-protein diets or incorporated a prior weight-loss period. This issue is, to some extent, semantic given that since 90% of people with T2D are obese in Western countries (117), there is a rationale for high-protein diets as a weight-loss strategy to improve glycemic control. However, whether this represents an effective longer-term approach remains to be established.

A number of studies have investigated the effects of high-protein diets on glycemic variability, which has recently emerged as a target for glycemic control and, potentially, an independent risk factor for the micro- and macrovascular complications of T2D, particularly when glycemic control is ‘reasonable’ (i.e. HbA_1c_ ≤8.0%) (113, 118–122). For example, in 16 well-controlled T2D patients, replacing an isocaloric standard-protein (16% protein) with a high-protein (29% protein), diet, for two separate 48-hour periods, was associated with reductions in indices of glycemic variability by 34 to 45%, supporting the concept that a higher intake of protein should be incorporated in dietary advice for patients with T2D (120). Comparable effects were also observed among 20 insulin-resistant women with obesity, where a high-protein diet was more effective in reducing glycemic variability, compared with a Mediterranean diet, in a 21-day trial (121). Furthermore, in a study by Fabricatore et al., in which 26 participants with obesity and T2D underwent a 3-day continuous glucose monitoring (CGM), a higher protein intake was associated with reduced glycemic variability (122). While these findings are promising, confirmation in longer-term studies is required before recommending changes to clinical practice.

A lesser number of studies have examined the effects of selected animal- and plant-based protein sources on weight and glycemia, again with inconsistent outcomes (123–136) (**Table 2**). While these studies have focused primarily on weight loss, rather than glycemia, observed glucoregulatory effects of different protein sources were in the majority of these studies found not to differ. For example, when the effects of supplemental whey and soy protein (~56 g/d) were compared to an isoenergetic amount of carbohydrate among free-living overweight and obese participants, slightly, but significantly, greater weight loss was observed with whey (-1.8 kg), compared with soy protein (-0.9 kg), with no differences in their effects on fasting glucose (123). In another study of 48 participants with obesity, the effects of two formulas containing either soy (12 g) and milk proteins (9 g) or only milk protein (22 g), given daily every morning for 20 weeks, were compared, and milk protein (-2.5 kg) had superior effects in inducing weight loss than soy protein (-1.1 kg), and also led to a greater reduction in HbA_1c_ levels (124). Another study reported that milk induced a greater reduction in body weight (-4.43 kg) over a period of 8 weeks compared with calcium-fortified soy milk (-3.46 kg) (126). In contrast, greater weight loss effects were reported after 12 weeks with soy- (-9%), rather than milk-based (-7.9%), meal replacements within an energy-restricted diet (125). Interestingly, reductions in fasting glucose were only evident with the soy-, but not with milk-based, meals in this study (125). Consumption of either 3 soy, or 3 casein, shakes daily as part of a 16-week, energy-restricted diet, in two groups of women with obesity, had comparable effects on weight loss and body composition, as well as fasting insulin, while a greater reduction in fasting glucose was evident in the soy group (127). In another study, no difference was found between the weight-reducing effects of a meat-based (-2.2 kg), and a soy-based (-2.4 kg), diet (~30% of energy from protein), with a significant reduction in fasting glucose observed with both diets (130). Two studies reported that beef and chicken, as the primary sources of protein in an energy-restricted diet, had comparable weight loss effects (128, 129). Abete et al. reported that an energy-restricted diet with a high content of legumes (consumed 4 days per week with 17% protein from energy intake) led to body weight reductions comparable to those achieved with a high-protein diet (30% protein) mainly composed of animal proteins, which was associated with significantly greater reduction in fasting glucose only in legume diet group (131). Altogether, there is, therefore, no compelling evidence that a particular protein source leads to greater weight loss, or improvement in glycemia, than another, indicating that plant-based proteins are likely to be as effective for weight loss as animal-based proteins. Moreover, in some cases, plant-based sources were associated with more potent positive glucoregulatory effects (125, 127, 131). Importantly, it remains uncertain whether the effects of the source of protein are independent of other macro- and micronutrient contents.

**Table 2:** Intermediate-term effects of different protein sources (animal vs. plant) on energy intake, body weight and glycemia.

First author	Country	Design	Sample size (n)	Age (y)	BMI (kg/m^2^)	Health status	Protein source	Protein dose^1^	Duration^2^ (week)	EI^3^ (kcal/d)	Weight loss^4^	Glycemia^5^	Ref
Baer et al. (2011)	USA	Parallel	48 (M/F)	49-53	31	Overweight and obesity	Soy Whey	56 g/d	23	2,268 2,184	0.9 kg 1.8 kg	0.255* 0.255	(123)
Takahira et al. (2011)	Japan	Parallel	48 (M/F)	54-57	29	Obesity with/without T2D	Soy Milk	12 g/d SP+9 g/d MP 22 g/d MP	20	1,719 1,799	1.1 kg 2.5 kg	-0.9%** -1.4%	(124)
Anderson et al. (2005)	USA	Parallel	52 (M/F)	46-47	34	Overweight and obesity	Soy Milk	18 g/d 13 g/d	12	NR	9.0%* 7.9%	-3.6%*** -2%	(125)
Faghih et al (2011)	Iran	Parallel	43 (F)	37-38	31	Overweight and obesity	Calcium-fortified soy milk Milk	18% of EI (~57 g/d)	8	1,280 1,297	3.6 kg 4.4 kg	NR	(126)
Anderson et al. (2007)	USA	Parallel	43 (F)	44-46	35	Obesity	Soy Casein	67.2 g/d 62.1 g/d	16	NR	11.9 kg 13.4 kg	5.1 5.2	(127)
Mahon et al. (2007)	USA	Parallel	54 (F)	58	29.6	Obesity with prediabetes	Non-meat protein-based diet Chicken-based diet Beef-based diet	50 g/d 80 g/d 80 g/d	9	1,158 1,098 1,114	5.6 kg 7.9 kg 6.6 kg	5.6 5.6 5.6	(128)
Neacsu et al. (2014)	UKD	Cross-over	20 (M)	51	34.8	Obesity	Soy-based diet Meat-based diet	153 g/d 154 g/d	2	2,072 2,098	2.4 kg 2.2 kg	5.4 5.3	(130)
Abete et al. (2009)	Spain	Parallel	25 (M)	38	31.8	Obesity	Legume-based diet High-protein diet	17% of EI (~74 g/d) 30% of EI (~137 g/d)	8	1,537 1,765	8.3 kg 8.6 kg	-5.1%*** -4.2%	(131)
Melanson et al. (2003)	USA	Parallel	61 (F)	43	32.1	Overweight	Chicken-based diet Beef-based diet	76.3 g/d 72 g/d	12	NR	6 kg 5.6 kg	NR	(129)
Aldrich et al. (2011)	USA	Parallel	12 (M/F)	50	30	Overweight	Mixed protein-diet Whey protein-diet	124 g/d	20	NR	7.6 kg 9.6 kg	NR	(132)

M, Male; F, Female; Y, Years; BMI, Body mass index; EI, Energy intake; T2D, type 2 diabetes; SP, Soy protein; MP, Milk protein; NR, Not reported.

^1^ Reported as grams per day intake of protein, otherwise indicated as percentage of daily energy intake (*); ^2^Intervention duration; ^3^ Reported as daily energy intake (kcal); ^4^Reported as changes in body weight (kg), otherwise indicated as percentage of weight change (*); ^5^ Reported as fasting glucose levels in mmol/L (or log mmol/L*), otherwise indicated as changes in either HbA1c levels% (**) or fasting glucose levels (***).

## 4 Longer-term effects of dietary protein intake in obesity and T2D

**Figure 2:**
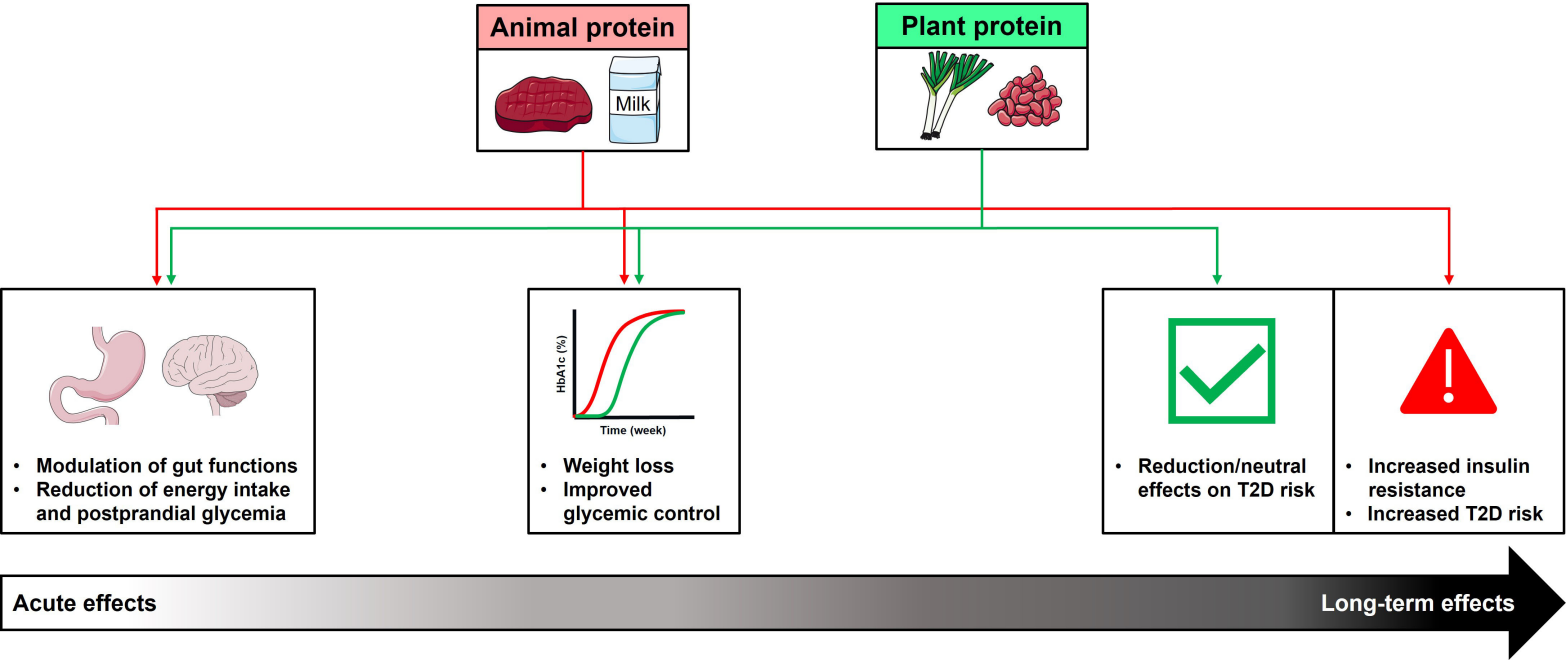
Summary of acute, intermediate-, and longer-term effects of dietary animal and plant protein consumption on metabolic health. Acute intakes of protein (animal or plant-based protein), either in an isolated form as ‘preloads’, or as part of a meal, stimulate GI hormones, associated with reductions in energy intake and postprandial blood glucose. These acute effects are associated with greater weight loss, and improved glycemic control, when consumed as part of a high-protein diet, with comparable outcomes observed with both types of protein. In contrast, the outcomes of longer-term studies suggest that long-term consumption of animal-based protein may have adverse effects in relation to the development of type 2 diabetes (T2D), while plant-based protein have either protective or neutral effects. This may reflect animal protein-specific effects to increase insulin resistance, leading to increased risk of T2D.

While protein has, for many years, represented the cornerstone of dietary approaches for weight management in obesity, associated with improved glycemic control, there is a lack of consensus regarding the maximal amount of dietary protein that can be consumed in the long-term without adverse effects. Interestingly, in contrast to the beneficial acute and intermediate-term effects of protein on weight loss and glycemic control discussed above, outcomes of large prospective studies investigating the association between the long-term consumption of protein with body weight and/or T2D have indicated no overall beneficial effects (137–139). Moreover, there is evidence that the long-term health effects of protein may vary according to the source of protein, thus, long-term consumption of animal-based proteins may have adverse effects in relation to obesity and T2D, while plant-based proteins have either protective or neutral effects (**Figure 2**).

Several epidemiological studies, investigating the role of greater protein intake from different sources in the development of obesity in large populations, have consistently reported a direct association between prospective weight gain and higher animal protein intake, and by inference, the risk of obesity (137, 140–142) (**Table 3**). For example, in the European Prospective Investigation into Cancer and Nutrition (EPIC) study, of 89,432 weight-stable men and women from five countries, overall associations were evident between higher daily intakes of total and animal protein and subsequent weight gain over 6.5 years, which was mainly attributed to protein derived from red and processed meats and chicken, rather than to fish and dairy products (137). In contrast, there were neither protective, nor adverse, associations with plant-based proteins (137). A 2015 analysis, examining the relationships between consumption of different protein sources with long-term weight gain across three separate prospective cohorts of US men and women (the Nurses’ Health Studies (NHS) I and II and the Health Professionals Follow-Up Study (HPFS)) revealed that animal-based protein sources were independently associated with long-term weight gain (i.e. each increased serving/day of red meat, chicken and regular cheese was associated with a 0.13–1.17 kg weight gain), whereas plant-based proteins were independently associated with relative weight loss (i.e. each increased serving/day of peanut butter, walnuts or other nuts was associated with -0.14 to -0.71 kg weight loss) over 4 years (140). Similarly, in a recent analysis of the NHS II study, over a 26-year follow-up, intakes of red meat (both fresh and processed products) and high-fat dairy products, were associated with an increased risk of nonalcoholic fatty liver disease (NAFLD), with obesity found to be the major contributor, while a higher intake of nuts was associated with a reduced risk (143). In another cohort of 1,730 employed men, aged 40 to 55 years from the Chicago Western Electric Study, which were followed from 1958 to 1966, animal protein was positively associated with a 4 times greater risk of obesity, while plant protein reduced the risk by 50% (141). Recent studies have also found that substituting different animal protein sources, particularly processed red meats, with plant protein was associated with reduced risks of coronary heart disease (CHD) and all-cause mortality (144–146).

**Table 3:** Long-term effects of total, animal and plant protein intake on body weight.

First author	Country	Design	Sample size (n)	Age (y)	BMI (kg/m^2^)	Protein source	Protein dose^1^	Duration^2^ (y)	Weight change^3^	Ref
Halkjær et al. (2011)	European countries	Prospective cohort	89,432 (M/F)	35-65	20-33	Total protein Animal protein Plant protein	Per increased 150 kcal/d	6.5	+0.052 kg/year (+0.025 to +0.079) +0.056 kg/year (+0.026 to +0.085) +0.017 kg/year (-0.032 to +0.068)	(137)
Bujnowski et al. (2011)	USA	Prospective cohort	1,730 (M)	40-55	24->30	Total protein Animal protein Plant protein	17.2 vs. 13% of EI* 13.8 vs. 9.3% 4.1 vs. 2.9%	7	3.27 (1.94 to 5.51)* 4.62 (2.68 to 7.98) 0.58 (0.36 to 0.95)	(141)
Smith et al. (2015)	USA	Prospective cohort	120,784 (M/F)	30-50	22->25	Animal protein Plant protein	Per increased serving/d	16-24	+0.13 to +1.17 kg/4 years -0.14 to -0.71 kg/4 years	(140)

M, Male; F, Female; Y, Years; BMI, Body mass index; EI, Energy intake.

^1^ Reported as grams per day intake of protein, otherwise indicated as percentage of daily energy intake (*); ^2^ Follow up duration; ^3^ Reported as either body weight change (kg), otherwise indicated as risk of obesity (*).

The majority of studies have also reported that long-term consumption of animal protein increased the risk of T2D, while plant proteins had protective or neutral effects (34–36, 147–154) (**Table 4**). For example, in two large cohort studies (Women’s Health Initiative and the UK Biobank), with 16,505 incident cases of T2D (out of 143,297 adults without T2D at baseline), during a median follow-up of 15.8 years, replacing consumption of animal protein (5% of energy intake) with plant protein was associated with a 21% lower risk of T2D, attributable to reductions in obesity-related inflammatory factors (36). Moreover, in another study, a higher intake of animal, but not plant, protein was associated with increased risks of both prediabetes and T2D; so that each 5% increment in energy intake from animal protein at the expense of carbohydrate was associated with increased risks of prediabetes of 35% and T2D of 37% (154). This was attributable primarily to increased insulin resistance, as assessed by the indirect homeostatic model (HOMA-IR) (154). A number of recent reviews and meta-analyses have also concluded that higher animal, but not plant, protein intake is associated with an increased risk of T2D (155–160).

**Table 4:** Long-term effects of total, animal and plant protein intake on glycemic control.

First author	Country	Design	Sample size (n)	Age (y)	BMI (kg/m^2^)	Protein source	Protein dose^1^	Duration^2^ (y)	T2D cases (n)	T2D risk	Ref
Song et al. (2004)	USA	Prospective cohort	37,309 (F)	>45	20->30	Animal protein Plant protein	77 vs. 40 g/d 37 vs. 17 g/d	8.8	1,558	1.44 (1.16 to 1.78) 0.85 (0.70 to 1.03)	(150)
Sluijs et al. (2010)	The Netherlands	Prospective cohort	38,094 (M/F)	21-70	24->30	Total protein Animal protein Plant protein	Per increased 10 g/d	10.1	918	1.16 (1.06 to 1.26) 1.13 (1.04 to 1.22) 1.04 (0.83 to 1.29)	(147)
van Nielen et al. (2014)	European countries	Prospective cohort	26,253 (M/F)	53 (mean)	<25- >30	Total protein Animal protein Plant protein	Per 10 g/d	12	11,637	1.06 (1.02 to 1.09) 1.05 (1.02 to 1.08) 1.04 (0.93 to 1.16)	(152)
Malik et al. (2016)	USA	Prospective cohort	205,802 (M/F)	30-50	22->25	Total protein Animal protein Plant protein	22 vs. 14% of EI* 17 vs. 9% 7 vs. 4%	20.1	15,580	1.07 (1.01 to 1.17) 1.13 (1.06 to 1.21) 0.91 (0.84 to 0.98)	(34)
Shang et al. (2016)	AUS	Prospective cohort	21,523 (M/F)	27-80	20->30	Total protein Animal protein Plant protein	Per 5% of EI*	11.7	929	1.15 (1.00 to 1.32) 1.15 (1.00 to 1.33) 1.00 (0.69 to 1.46)	(149)
Sugihiro et al. (2019)	USA	Prospective cohort	765 (M/F)	58.1 (mean)	≥25	Total protein Animal protein Plant protein	Per 1% of EI*	10.7	36	1.22 (1.03 to 1.45) 1.20 (1.04 to 1.38) 0.82 (0.62 to 1.09)	(151)
Chen et al. (2020)	The Netherlands	Prospective cohort	6,813 (M/F)	≥45	–	Total protein Animal protein Plant protein	Per 5% of EI*	7.2	643	1.37 (1.18 to 1.58) 1.37 (1.19 to 1.58) 1.21 (0.83 to 1.77)	(154)
Yuan et al. (2021)	China	Prospective cohort	7,312 (M/F)	48.3 (mean)	22->25	Total protein Animal protein Plant protein	92 vs. 41 g/d 41 vs. 3 g/d 65 vs. 25 g/d	5.8	209	2.38 (1.43 to 3.98) 1.93 (1.17 to 3.17) 1.20 (0.71 to 2.04)	(153)
Li et al. (2022)	USA UKD	Prospective cohort	108,681 (F) 34,616 (F)	50-80	22->30	Total protein Animal protein Plant protein Total protein	86 vs. 50 g/d 67 vs. 28 g/d 28 vs. 13 g/d Per 5% of EI*	15.8 11.4	15,842 663	1.24 (1.18 to 1.30) 1.31 (1.24 to 1.37) 0.82 (0.78 to 0.86) 1.14 (0.99-1.32)	(36)

M, Male; F, Female; Y, Years; BMI, Body mass index; T2D, Type 2 diabetes; EI, energy intake.

^1^ Reported as grams per day intake of protein, otherwise indicated as percentage of daily energy intake (*); ^2^ Follow up duration.

The mechanisms through which a high protein intake, from animal vs. plant-based sources, may have differential impacts in the long-term are poorly defined. However, there are several potential explanations, including differences in amino acid composition, glycemic load and potential deleterious effects of the high insulinotropic properties of animal protein, which, in turn, promotes fat storage and impedes fat oxidation (161, 162). Preclinical models also indicate that increased levels of specific amino acids, particularly BCAAs, which are abundant in animal-based proteins, could lead to insulin resistance by activating mTOR, to initiate a detrimental feedback loop toward insulin receptor substrate 1 signaling (163, 164). Indeed, insulin-mediated glucose uptake decreases when body tissues are chronically overexposed to high levels of insulin. Thus, prolonged hyperinsulinemia may lead to insulin resistance and, ultimately, T2D. Elevated postprandial levels of BCAAs have also been shown to inhibit muscle glucose transport and/or glucose phosphorylation directly, to reduce glycogen synthesis, further contributing to insulin resistance (165). Limited human studies also indicate that an increase in protein intake in the longer-term can reduce insulin sensitivity (166, 167). For example, in healthy participants, a higher consumption of protein (~1.87 g/kg of body weight) for six months was associated with greater glucose-stimulated insulin secretion and a modest reduction in insulin sensitivity (166). In another study, in overweight participants, comparing an isoenergetic high-protein diet (~25–30% protein) with a conventional-protein (~15% protein) control diet over 18 weeks, a reduction in insulin sensitivity, as measured by the euglycemic hyper-insulinemic clamp, was observed (167). The differential impacts of animal and plant protein may also be influenced by other dietary nutrients. For example, plant-based foods are rich in dietary fiber, which is known to mitigate T2D risk and may interact additively with plant protein (168). In contrast, a number of dietary components in red and processed meats, as the primary sources of animal protein, such as heme iron, animal fat, and advanced glycation end products, may be, both directly and indirectly, associated with an increased T2D risk. This association may reflect factors including obesity and its related inflammatory markers (leptin and endothelial dysfunction biomarkers) (36, 154, 169).

It is important to also appreciate other potential deleterious effects of high-protein diets, particularly increased risks of osteoporosis and renal diseases (170–174). A potential link with osteoporosis was supported by the observation of increased urinary calcium excretion during a high protein intake (170–172). High-protein diets (>2 g/kg/day) may also increase bone resorption by increasing the acid load in the body, compared with diets of low- to normal-protein content of 0.7-1.0 g/kg/day (172). Indeed, it has been suggested that high consumption of animal-based protein, in particular, leads to an acidification of the blood that may increase carbonate, and subsequently calcium, release from the skeleton to decrease bone mineral density (171). In an epidemiological study of older men (>60 years), a greater dietary acid load due to a chronic high-protein intake was associated with femoral bone loss only under conditions of very low calcium intake <800 mg/d dietary calcium (173). An increased renal acid load, such as the sulfuric acid produced from the oxidation of different amino acids, has also been suggested to increase the risk of kidney stones, and/or increase the glomerular filtration rate, which may lead to renal dysfunction over time (174). While these findings are yet to be confirmed in different populations, they further support that recommendations for a higher protein intake in the long-term should be circumspect.

## 5 Conclusions and recommendations/priorities for future studies

There is strong evidence from short-term studies (i.e. <6 months in duration) that a higher dietary protein intake facilitates weight loss in obesity and improves glycemic control in T2D. In contrast, the outcomes of longer-term studies, of which there are less, are not convincing, precluding clear-cut recommendations. Suboptimal dietary adherence and metabolic adaptations are likely to contribute to this apparent anomaly, as well as methodological limitations with respect to the type and duration of studies, characteristics of study participants, and how well-controlled the studies are. While acute studies are well-controlled and provide the most reliable findings, these are characteristically performed among a smaller number of participants, who are predominantly young. Accordingly, longer-term studies with larger and more heterogeneous populations are required. An important issue, which has received inappropriately little attention, is the source of dietary protein (i.e. animal vs. plant). The importance of this issue is highlighted by recent epidemiological studies, which strongly support the concept that animal-, but not plant-, based protein intake may have adverse effects in relation to the development of obesity and T2D. Importantly, the longer-term comparative effects of high-protein diets, based on different sources, on body weight and glycemic control remain to be formally evaluated. Despite this limitation, it would be appropriate for current dietary guidelines to consider the source of dietary protein in relation to the use of high-protein diets, and reasonable to advise a reduction in the consumption of animal protein and a relatively increased intake of plant protein. Such a nuanced approach may prove fundamental to longer-term outcomes. Moreover, future studies should focus on the relevance of animal vs. plant-based protein sources, particularly how longer-term consumption of different protein sources may affect GI-related food intake- and gluco-regulatory mechanisms. The outcomes of such studies are likely to lead to more personalized and effective use of protein in the prevention and management of obesity and T2D.

## Author contributions

JA-S: Writing – review & editing, Writing – original draft, Conceptualization. CF-B: Writing – review & editing, Conceptualization. MH: Writing – review & editing, Conceptualization.
